# HIV Stigma and Viral Suppression Among People Living With HIV in the Context of Universal Test and Treat: Analysis of Data From the HPTN 071 (PopART) Trial in Zambia and South Africa

**DOI:** 10.1097/QAI.0000000000002504

**Published:** 2020-09-24

**Authors:** James R. Hargreaves, Triantafyllos Pliakas, Graeme Hoddinott, Tila Mainga, Constance Mubekapi-Musadaidzwa, Deborah Donnell, Estelle Piwowar-Manning, Yaw Agyei, Nomhle F. Mandla, Rory Dunbar, David Macleod, Sian Floyd, Peter Bock, Sarah Fidler, Richard J. Hayes, Janet Seeley, Anne Stangl, Virginia Bond, Helen Ayles

**Affiliations:** a Department of Public Health, Environments and Society, London School of Hygiene and Tropical Medicine, London, United Kingdom;; b Desmond Tutu TB Centre, Department of Paediatrics and Child Health, Faculty of Medicine and Health Sciences, Stellenbosch University, Cape Town, South Africa;; c Zambart, School of Medicine, University of Zambia, Lusaka, Zambia;; d SCHARP, Seattle, WA;; e Johns Hopkins University School of Medicine, Baltimore, MD;; f Imperial College NIHR BRC, Imperial College London, London, United Kingdom; and; g International Center for Research on Women, Washington, DC.

**Keywords:** HIV stigma, viral suppression, PLHIV, community members, health workers

## Abstract

**Background:**

The impact of HIV stigma on viral suppression among people living with HIV (PLHIV) is not well characterized.

**Setting:**

Twenty-one communities in Zambia and South Africa, nested within the HPTN 071 (PopART) trial.

**Methods:**

We analyzed data on viral suppression (<400 copies HIV RNA/mL) among 5662 laboratory-confirmed PLHIV aged 18–44 years who were randomly sampled within the PopART trial population cohort 24 months after enrolment (PC24). We collected data on experiences and internalization of stigma from those PLHIV who self-reported their HIV status (n = 3963/5662) and data on perceptions of stigma from a 20% random sample of all PLHIV (n = 1154/5662). We also measured stigma at the community-level among PLHIV, community members, and health workers. We analyzed the association between individual- and community-level measures of HIV stigma and viral suppression among PLHIV, adjusting for confounding.

**Results:**

Of all 5662 PLHIV, 69.1% were virally suppressed at PC24. Viral suppression was highest among those 3963 cohort participants who self-reported living with HIV and were on ART (88.3%), and lower among those not on treatment (37.5%). Self-identifying PLHIV who reported internalized stigma were less likely to be virally suppressed (75.0%) than those who did not (80.7%; adjusted risk ratio, 0.94 95% CI: 0.89 to 0.98). Experiences, perceptions, and community-level measures of stigma were not associated with viral suppression.

**Conclusion:**

Internalized stigma among PLHIV was associated with a lower level of viral suppression; other dimensions of stigma were not. Stigma reduction approaches that address internalized stigma should be an integral component of efforts to control the HIV epidemic.

## Abstract 2

Supplemental Digital Content is Available in the Text.

## INTRODUCTION

Increasing the proportion of people living with HIV (PLHIV) who are virally suppressed is critical.^1^ UNAIDS 90:90:90 targets reflect the cascade steps needed to achieve this: diagnosis of PLHIV, rapid initiation of antiretroviral treatment (ART), and adherence to treatment to sustain viral suppression. Although most sub-Saharan African countries have implemented “treat all” policies, under which PLHIV are offered ART regardless of CD4 count, progress is suboptimal. In 2019, UNAIDS estimated that viral suppression among PLHIV was 92% in eSwatini, 72% in Malawi, 69% in Tanzania, and 77% in Zambia. In South Africa, only 64% of all PLHIV are virally suppressed,^2^ the country accounting for approximately 20% of the global burden.

We hypothesize that HIV stigma may act as a barrier to PLHIV achieving viral suppression and that this effect may arise because stigma can reduce PLHIV being diagnosed quickly, slow the rate at which PLHIV start treatment, and/or impede antiretroviral medication adherence and attendance for follow-up ART collections and retention in care. Access to HIV testing has been constrained by stigma among men who have sex with men in high-income settings,^3^ and among the general population in low-income settings,^4,5^ because clients feel shame in accessing testing, anticipate being stigmatized when accessing services,^6^ or want to avoid being associated with HIV. For similar reasons, stigma acts as a barrier to PLHIV accessing care in the United States,^7^ among pregnant women who test positive in antenatal settings in Africa,^8^ and more generally in low- and middle-income settings.^9^ Adherence to antiretroviral medication is lower in the presence of stigma among young people in the United States^10^ and globally^11^ because stigma negatively affects mental health and prevents PLHIV from disclosing their status to others and accessing psychosocial support.^6^

We therefore investigated the association between HIV stigma and viral suppression among a large, representative sample of PLHIV in the 21 communities participating in the HPTN 071 (PopART) trial in Zambia and South Africa.^12,13^ At 24 months (PC24), we assessed (1) whether PLHIV who reported experienced, internalized, or perceived stigma had lower rates of viral suppression, (2) whether PLHIV living in communities with higher levels of stigma had lower rates of viral suppression among PLHIV, and, (3) whether associations differed between the arms of the trial, which delivered different approaches to HIV testing and treatment. We focused on viral suppression as our primary outcome and hypothesized that stigma would reduce viral suppression as a result of the negative effect of stigma on each step in the HIV-care cascade. Thus, we show associations between stigma and self-reported (SR) treatment access and adherence as supporting analyses.

## METHODS

### Setting

The HPTN 071 (PopART) trial was a 3-arm cluster randomized trial conducted between 2013 and 2018 in 21 urban/periurban study communities (12 in Zambia and 9 in Western Cape Province, South Africa).^12,14^ Study communities were arranged in 7 triplets matched on geographical location and estimated HIV prevalence. Study communities in each triplet were randomly allocated to 3 study arms. In the 2 treatment arms (A and B), a new cadre of community-based health workers (HWs) known as Community HIV care Providers (CHiPs) carried out door-to-door HIV testing and referral services. In arm A, ART was offered to PLHIV regardless of CD4 count from the start of the trial; in arms B and C, ART was offered according to national guidelines, which changed over the course of the trial and became regardless of CD4 count in 2016. At PC24, viral suppression among PLHIV was higher, and HIV incidence was 20% lower in arms A and B than in arm C.^12^ In all arms, health facility-based and existing community-based HWs received training on the study aims but did not receive specific antistigma training.

### Outcome Study Population

The main study population for this analysis was PLHIV recruited within a population-based cohort (PC). In each community, 1 randomly selected adult aged 18–44 years was selected from each of a random sample of households. Enrolment mostly occurred between December 2013 and March 2015, although some additional participants were enrolled later in some communities. PC participants were surveyed at baseline (PC0) and at 12, 24, and 36 months (PC12/PC24/PC36; no data from PC36 were used in this analysis). Blood samples were drawn, and HIV viral load testing was performed on all laboratory-confirmed HIV-positive participants at PC24.

**FIGURE 1.**
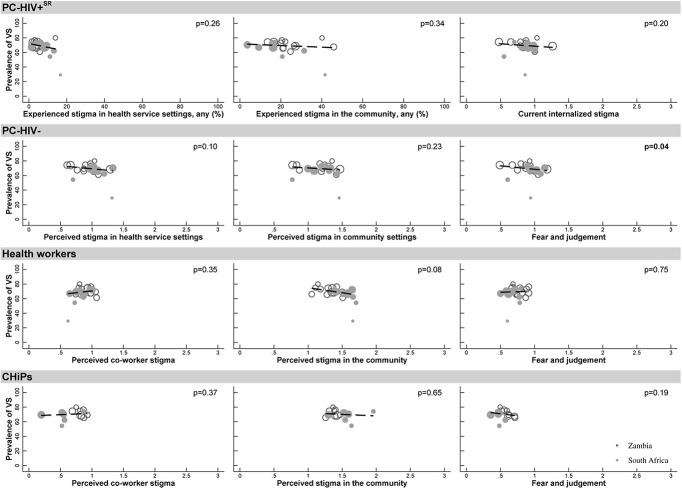
The association between the beliefs and perceptions of PC-HIV+^SR^, PC-HIV, HWs, and CHiPs and levels of viral suppression among 6233 laboratory-confirmed HIV-positive participants in 21 communities in South Africa and Zambia.

For this analysis, we excluded individuals with missing data on viral load, critical sociodemographic factors, SR HIV status, testing and treatment history, or HIV stigma at PC24, such that laboratory-confirmed HIV-positive 5662 participants were included (see Figure 1, Supplemental Digital Content, http://links.lww.com/QAI/B538). We refer to this group as PC-HIV+ throughout the article. The 571 individuals (9.2%) who had incomplete data showed no significant differences in sex, age, or viral suppression from the 5662 PC-HIV+ participants (see Table 1, Supplemental Digital Content, http://links.lww.com/QAI/B538).

Questions on experienced and internalized stigma could only be asked of participants who SR living with HIV (n = 3963). We refer to this sub-group as PC-HIV+^SR^. Additionally, a 20% subsample of all PC-HIV+ participants (regardless of SR HIV status) were randomly selected to answer additional questions on perceived stigma (n = 1154). We refer to this subgroup as PC-HIV+^sub^.

Blood samples were analyzed in-country using a single fourth-generation assay (Architect HIV Ag/Ab Combo Assay; Abbott Diagnostics, Delkenheim, Germany). Further testing was performed at the HPTN Laboratory Center (Johns Hopkins University, Baltimore, MD). Samples that had reactive results in-country were tested with a second fourth-generation assay (GS HIV Combo Assay; Bio-Rad Laboratories, Redmond, WA). HIV viral load testing was performed at the HPTN Laboratory Center (assay cut-off 400 copies/mL) for all PC-HIV+ participants at PC24 to determine their viral suppression status (not virally suppressed/virally suppressed), and we used this binary variable as our outcome for analysis.

### Measurement of Stigma Exposures

For individual-level stigma exposures among PC-HIV+^SR^, we used 4 measures reflecting experienced stigma in the community (combining 5 items), experienced stigma in health service settings (3 items), internalized stigma (3 items), and a combined measure of any type of stigma (11 items).^15^ Among PC-HIV+^sub^, we used 2 measures reflecting perceived stigma in the community (5 items) and perceived stigma in health service settings (2 items). Item wording is presented in Table 1. All measures used in the study had been previously validated, and Cronbach alpha and other measurement details for each measure are shown in Table 2, Supplemental Digital Content, http://links.lww.com/QAI/B538.^15^

**TABLE 1.** Description of Stigma Exposure Variables

Population Group and Exposure	Stigma Items/Statements
PC-HIV+^SR^	
Any reported internalized stigma (3 items)	(1) I have lost respect or standing in the community because of my HIV status, (2) I think less of myself because of my HIV status, (3) I have felt ashamed because of my HIV status
Any reported experienced stigma in the community (5 items)	(1) People have talked badly about me because of my HIV status, (2) someone else disclosed my HIV status without my permission, (3) I have been verbally insulted, harassed, and/or threatened because of my HIV status, (4) I have been physically assaulted because of my HIV status, (5) I have felt that people have not wanted to sit next to me, eg, on public transport, at church, or in a waiting room because of my HIV status
Any reported experienced stigma in health service settings (3 items)	(1) I have been denied health services because of my HIV status, (2) healthcare workers talked badly about me because of my HIV status, (3) a health worker disclosed my HIV status without my permission
Any stigma (11 items)	All items above
PC-HIV− and PC-HIV+	
Any negative attitudes (fear and judgment using 3 items)	(1) I fear that I could contract HIV if I come into contact with the saliva of a person living with HIV, (2) I would not like to sit close to someone living with HIV, eg, on public transport, at church, or in a waiting room, (3) I would be ashamed if someone in my family had HIV
PC-HIV− only	
Any perceived stigma in community settings (5 items)	(1) People thought to be living with HIV are sometimes physically assaulted, (2) people sometimes talk badly about PLHIV to others, (3) people thought to be living with HIV lose respect or standing, (4) people thought to be living with HIV are verbally insulted, harassed and/or threatened, (5) people sometimes disclose that other people are HIV positive without their permission
Any perceived stigma in health service settings (2 items)	(1) Health workers sometimes talk badly about people living with or thought to be living with HIV to others, (2) health workers sometimes disclose that other people are HIV positive without their permission
HW and CHiPs	
Any negative attitudes (fear and judgment using 5 items)	(1) I fear that I could contract HIV if I come into contact with the saliva of a person living with HIV, (2) I avoid physical contact with clients living with HIV, (3) HIV is punishment from God, (4) other people deserve access to health services more than PLHIV, (5) I would be ashamed if someone in my family had HIV
HW-HIV- only	
Any perceived stigma in the community (5 items)	(1) People thought to be living with HIV are sometimes physically assaulted, (2) people sometimes talk badly about PLHIV to others, (3) people thought to be living with HIV lose respect or standing, (4) people thought to be living with HIV are verbally insulted, harassed, or threatened, (5) people hesitate to start ARV drugs because they are afraid others will learn they are living with HIV
Any perceived coworker stigma (4 items)	(1) My coworkers sometimes talk badly about people thought to be living with HIV, (2) my coworkers sometimes gossip about clients' HIV test results, (3) my coworkers sometimes treat PLHIV poorly when providing them with health services, (4) my coworkers sometimes verbally insult clients living with HIV

In addition to individual-level analysis, we also developed community-level measures of stigma. To create these measures, we used data from 2 further study populations. First, in each community, we recruited a random 20% of PC participants who did not self-report living with HIV and were confirmed as tested HIV negative. We refer to this population as PC-HIV−. Data from this group were used to characterize the communities (n = 4339 at PC24). We developed 3 stigma measures reflecting average levels of fear and judgment (3 items), perceived stigma in the community (5 items), and perceived stigma in the health service setting (2 items). Community-level scores had a theoretical range from 0 to 3 such that, for example, a mean score of 1 indicated that people in that community on average responded “disagree” to stigma items and a mean score of 2 indicates people who on average responded “agree.” Details of the item wording and other measurement details are shown in Table 1 and Table 2, Supplemental Digital Content, http://links.lww.com/QAI/B538.

Second, we collected data from HWs, including the study-specific CHiPs workers in arms A and B, in a separate cohort study. Participants included CHiPs and HWs (in all trial Arms) who were enrolled between July 2014 and May 2015 [round 1 (R1)].^13^ CHiPs were eligible for the study upon recruitment to the PopART intervention team. HWs included doctors, nurses, laboratory technicians, cleaners, and security guards and community workers who worked within community HIV services on a regular (at least biweekly) basis. CHiPs and HWs were followed up in 2 rounds (R2 between June 2015 to June 2016 and R3 between January 2017 and February 2018).^16^ We generated community-level measures of stigma among HWs, using the same approach described above, reflecting the level of fear and judgment of PLHIV by CHIPs and HWs (including only data from those not self-reporting living with HIV, n = 516 and 1533, respectively, in R3) and perceptions of stigma in the community and by coworkers in the health facilities (including all individuals regardless of HIV status, n = 635 and 1802, respectively, in R3). Finally, for PC-HIV+^SR^, we also summarized the indicators used in individual analysis at the community level.

### Statistical Analysis

First, we described the study population of PC-HIV+, comparing characteristics between countries. We explored associations between stigma domains, as we had previously done at baseline.^17^

Second, we analyzed the association between sociodemographic, HIV testing and treatment characteristics, and viral suppression at PC24. We calculated risk ratios using modified Poisson regression with robust standard errors, adjusting for age, sex, and study community.

Third, we analyzed the individual-level association between HIV stigma measured among PC-HIV+^SR^ and PC-HIV+^sub^ and viral suppression at PC24, again calculating risk ratios adjusted for age, sex, and study community. We also calculated “fully” adjusted risk ratios using the same approach and additionally adjusting for marital status, education, and time of diagnosis (defined with 3 categories: before PopART, during PopART, and missing date).

Fourth, using interaction tests, we explored whether the strength of these associations differed by arm of the study.

Fifth, we produced cluster-level scatter plots to illustrate the strength of association between community-level measures of stigma, measured at either PC24 or round 3 of the health worker study, and the proportion of all PC-HIV+ who were virally suppressed at PC24. To quantify the strength of these associations, we used simple linear regression, weighted by the sample size in each community, and reported the *P* value for these associations.

Finally, in supplementary exploratory analyses, we recalculated both the individual-level and community-level stigma exposure variables using data from earlier rounds (PC0, PC12, and the health worker surveys at R1 and R2). We assessed whether there was evidence that the association between stigma and viral suppression at PC24 was similar using exposure measures from these earlier time points. In addition, we characterized internalized stigma as a continuous measure to assess whether the associations we identified were similar to our primary analysis where internalized stigma was included as a binary variable. We further report the association between internalized stigma and SR variables on the causal pathway to viral suppression: ever and currently taking ART, hiding pills, and ART adherence.

### Ethical Considerations

Ethical approval for all study procedures was obtained from the institutional review boards of the London School of Hygiene and Tropical Medicine, Stellenbosch University, and the University of Zambia. All participants provided written informed consent before enrolment.

## RESULTS

The 5662 PC-HIV+ participants were predominantly women (86.0%) and more were from Zambia (n = 3491) than South Africa (n = 2171) (Table 2). More participants had completed secondary education and were unmarried in South Africa than in Zambia. At PC24, only 4.4% of participants SR never having tested for HIV. However, 9.8% said that they did not know their current HIV status, and 15.9% SR that their status was HIV negative, with both figures higher in South Africa than in Zambia. Overall, 70.0% of PC-HIV+ reported that they knew their HIV-positive status, and of these, the majority reported that they were currently taking ART (63.0% of all PC-HIV+ in Zambia, 50.6% in South Africa); 69.1% of all PC-HIV+ had viral suppression.

**TABLE 2.** Characteristics of PC-HIV+ With Viral Suppression Data at PC24 in 21 Communities in Zambia and South Africa (n = 5662)

	Categories	Zambia		South Africa		Total	
		N	%	N	%	N	%
Sociodemographics							
Sex	Male	500	14.3	291	13.4	791	14.0
	Female	2991	85.7	1880	86.6	4871	86.0
Age groups, yr	18–24	732	21.0	373	17.2	1105	19.5
	25–34	1515	43.4	1012	46.6	2527	44.6
	35–44	1244	35.6	786	36.2	2030	35.9
Education groups	Did not complete secondary	1522	43.6	340	15.7	1862	32.9
	Completed secondary	1803	51.6	1757	80.9	3560	62.9
	Further	166	4.8	74	3.4	240	4.2
Marital status	Married	2092	59.9	713	32.8	2805	49.5
	Never married	421	12.1	1334	61.4	1755	31.0
	Divorced/separated	671	19.2	96	4.4	767	13.5
	Widowed	307	8.8	28	1.3	335	5.9
Study arm	Arm A	1194	34.2	709	32.7	1903	33.6
	Arm B	1094	31.3	655	30.2	1749	30.9
	Arm C	1203	34.5	807	37.2	2010	35.5

	Categories	Zambia		South Africa		Total	
		N	%	N	%	N	%
Test and Treatment Status							
Viral suppression status	Not suppressed	1029	29.5	720	33.2	1749	30.9
	Suppressed	2462	70.5	1451	66.8	3913	69.1
SR HIV testing and treatment status	Last test HIV positive on ART	2199	63.0	1098	50.6	3297	58.2
	Last test HIV positive but not currently on ART or with no data on ART	358	10.3	308	14.2	666	11.8
	Last test HIV negative	507	14.5	391	18.0	898	15.9
	Don't know HIV status	282	8.1	272	12.5	554	9.8
	Never tested	145	4.2	102	4.7	247	4.4
ART adherence (n = 3341)*	Yes	1938	87.8	967	85.4	2905	87.0
	No (poor adherence)	270	12.2	166	14.6	436	13.0
Time of diagnosis†	Before PopART	1286	50.3	902	64.2	2188	55.2
	During PopART	940	36.8	380	27.0	1320	33.3
	SR HIV+ with missing dates	331	12.9	124	8.8	455	11.5
	Did not self-report HIV positive	934		765		1699	

	Categories	Zambia		South Africa		Total	
		N	%	N	%	N	%
HIV Stigma							
Any stigma†	No stigma experienced	1680	65.7	1083	77.0	2763	69.7
	Any stigma experienced	877	34.3	323	23.0	1200	30.3
	Did not self-report HIV positive	934		765		1699	
Experienced stigma in health service settings (n = 3963)‡	Never	2442	95.5	1311	93.2	3753	94.7
	At least once	115	4.5	95	6.8	210	5.3
Experienced stigma in the community (n = 3963)‡	Never	1962	76.7	1182	84.1	3144	79.3
	At least once	595	23.3	224	15.9	819	20.7
Current internalized stigma (n = 3963)‡	Do not agree	2075	81.1	1255	89.3	3330	84.0
	Agree	482	18.9	151	10.7	633	16.0
Perceived stigma in health service settings (n = 1154)§	Do not agree	543	75.6	362	83.0	905	78.4
	Agree	175	24.4	74	17.0	249	21.6
Perceived stigma in the community (n = 1154)§	Do not agree	237	33.0	276	63.3	513	44.5
	Agree	481	67.0	160	36.7	641	55.5
Disclosed HIV status	No	272	10.6	234	16.6	506	12.8
	Yes	2285	89.4	1172	83.4	3457	87.2
	Did not self-report HIV positive	934		765		1699	
Have you ever hidden your ART pills?‖	No	1687	76.3	973	85.7	2660	79.5
	Yes	524	23.7	162	14.3	686	20.5
	Did not self-report HIV positive or ever start ART	1280		1036		2316	

* Poor or nonadherence was defined as “respondents self-reporting that they had ever started ART but were not currently taking ART or currently taking ART but had either stopped in the past 12 months, or missed pills in the past 7 days.”

† Time of first positive HIV test result was asked only to those who SR HIV positive.

‡ Stigma items captured by PLHIV who SR HIV positive.

§ Stigma items captured by PLHIV who completed the extended questionnaire (random 20% sample of the PC).

‖ Asked to everyone but those ever started on ART.

Of PC-HIV+^SR^, 30.3% reported having experienced any stigma, and this was higher in Zambia (34.3%) than in South Africa (23.0%). Stigma experienced in the community was the most common form (20.7%), followed by internalized stigma (16.0%) and stigma in a health setting (5.3%). Similar to our findings at baseline, most of those who reported stigma in a health setting also reported experiencing stigma in the community, and internalized stigma was more commonly reported by those who reported experienced stigma but was not associated with community-level stigma measures (see Table 3 and Figure 2, Supplemental Digital Content, http://links.lww.com/QAI/B538). Among PC-HIV+^sub^ participants, 67.0% of Zambians and 36.7% of South Africans reported that they perceived stigma to be present in communities, with lower proportions perceiving stigma in health service settings (24.7% and 17.7% respectively). Most PC-HIV+^SR^ had disclosed their status to someone, and a substantial minority (20.5%) of those who reported ever taking ART said that they had hidden their ART pills at some time (Table 2).

Viral suppression was lower in South Africa, among men, and among younger participants (Table 3). Viral suppression was highest among PC-HIV+ participants who reported currently taking ART (88.3%). Viral suppression was lower among those that did not answer questions on experience and internalization of stigma because they SR that they were HIV negative [adjusted risk ratio (aRR) = 0.48; 95% CI: 0.44 to 0.52], had never had an HIV test (aRR = 0.66; 95% CI: 0.59 to 0.73), or that they did not know their status (aRR = 0.54; 95% CI: 0.49 to 0.59). Viral suppression was also lower for those self-reporting poor adherence (aRR = 0.85; 95% CI: 0.81 to 0.90; Table 3) and for those self-reporting hiding their ART pills, although the statistical evidence for the latter was weak (aRR = 0.97; 95% CI: 0.94 to 1.00; Table 4).

**TABLE 3.** The Association Between Sociodemographic Characteristics, ART Adherence, and SR HIV Status With Viral Suppression Among at PC24 in 21 Communities in Zambia and South Africa (n = 5662)

Variable	Categories	% virally suppressed, n/N (%)	aRR* (95% CI)
Country†	Zambia	2462/3491 (70.5%)	1.00
	South Africa	1451/2171 (66.8%)	**0.93 (0.90 to 0.97)**
Age group‡	18–24	548/1105 (49.6%)	1.00
	25–34	1762/2527 (69.7%)	**1.41 (1.33 to 1.51)**
	35–44	1603/2030 (79.0%)	**1.61 (1.51 to 1.71)**
Sex	Male	458/791 (57.9%)	1.00
	Female	3455/4871 (70.9%)	**1.26 (1.18 to 1.33)**
ART adherence (n = 3341)§	Yes	2592/2905 (89.2%)	1.00
	No (poor adherence)	328/436 (75.2%)	0.85 (0.81 to 0.90)
Self-reported HIV testing and treatment status‖	Last test HIV positive on ART	2912/3297 (88.3%)	1.00
	Last test HIV positive but not currently on ART or with no data on ART	250/666 (37.5%)	**0.43 (0.39 to 0.48)**
	Last test HIV negative	355/898 (39.5%)	**0.48 (0.44 to 0.52)**
	Do not know HIV status	254/554 (45.8%)	**0.54 (0.49 to 0.59)**
	Never tested	142/247 (57.5%)	**0.66 (0.59 to 0.73)**

Bolded, *P* < 0.05

* Adjusted for age group, sex, and community with robust standard errors.

† The aRR is adjusted for age group and sex with robust standard errors.

‡ Overall *P* value for age group: *P* < 0.001.

§ Poor or nonadherence was defined as “respondents self-reporting that they had ever started ART but were not currently taking ART,or currently taking ART but had either stopped in the past 12 months, or missed pills in the past 7 days.”

‖ Overall *P* value for SR HIV testing and treatment status: *P* < 0.001.

n, number of individuals that are virally suppressed; N, total number of individuals within groups.

**TABLE 4.** The Association Between Experienced and Perceived HIV Stigma and Viral Suppression at PC24 in 21 Communities in Zambia and South Africa

Variable	Categories	n/N (%)	aRR* (95% CI)	aRR† (95% CI)
Self-report HIV status‡	SR HIV positive	3162/3963 (79.8%)	1.00	1.00
	Did not self-report HIV positive	751/1699 (44.2%)	**0.59 (0.56 to 0.63)**	**0.59 (0.56 to 0.63)**‡
Any stigma (n = 3963)	No stigma experienced	2223/2763 (80.5%)	1.00	1.00
	Any stigma experienced	939/1200 (78.3%)	0.97 (0.93 to 1.00)	0.97 (0.93 to 1.00)
Experienced stigma in health service settings (n = 3963)	Never	2995/3753 (79.8%)	1.00	1.00
	At least once	167/210 (79.5%)	1.00 (0.93 to 1.07)	0.99 (0.93 to 1.06)
Experienced stigma in the community (n = 3963)	Never	2512/3144 (79.9%)	1.00	1.00
	At least once	650/819 (79.4%)	0.98 (0.94 to 1.02)	0.98 (0.94 to 1.02)
Current internalized stigma (n = 3963)	Disagree	2687/3330 (80.7%)	1.00	1.00
	Agree	475/633 (75.0%)	**0.94 (0.89 to 0.98)**	**0.94 (0.89 to 0.98)**
Disclosed HIV status (n = 3963)	No	358/506 (70.8%)	1.00	1.00
	Yes	2804/3457 (81.1%)	**1.13 (1.06 to 1.20)**	**1.11 (1.05 to 1.18)**
	Did not self-report HIV positive	751/1699 (44.2%)	—	—
Have you ever hidden your ART pills? (n = 3963)	No	2334/2660 (87.7%)	1.00	1.00
	Yes	591/686 (86.2%)	0.97 (0.94 to 1.01)	0.97 (0.94 to 1.00)
	Self-report HIV positive but never started ART	237/617 (38.4%)	—	—
Population group receiving the extended stigma questionnaire	Not receiving the perceived stigma questions	3112/4508 (69.0%)	1.00	1.00
	Random 20% sample of the PC	801/1154 (69.4%)	1.00 (0.96 to 1.05)	1.01 (0.97 to 1.05)
Perceived stigma in health service settings (n = 1154)§	Do not agree	628/905 (69.4%)	1.00	1.00
	Agree	173/249 (69.5%)	1.05 (0.96 to 1.15)	1.05 (0.96 to 1.15)
Perceived stigma in the community (n = 1154)§	Do not agree	352/513 (68.6%)	1.00	1.00
	Agree	449/641 (70.0%)	1.03 (0.95 to 1.12)	1.01 (0.94 to 1.10)

Bolded, *P* < 0.05.

* Model adjusted for age group, sex, and community with robust standard errors.

† Model adjusted for age group, sex, education, marital status, time of diagnosis, and community with robust standard errors.

‡Model adjusted for age group, sex, education, marital status, and community with robust standard errors.

§ Stigma items captured by PLHIV who completed the extended questionnaire (random 20% sample of the PC).

n, number of individuals that are virally suppressed; N, total number of individuals within groups.

In unadjusted and fully adjusted analyses (Table 4), there was little evidence of an association between experienced stigma in either community or health service setting and viral suppression at PC24 among PC-HIV+^SR^. However, those reporting internalized stigma were less likely to be virally suppressed than those who did not (aRR = 0.94; 95% CI: 0.89 to 0.98). This association was similar in all 3 arms of the trial (see Figure 3, Supplemental Digital Content, http://links.lww.com/QAI/B538, interaction; *P* = 0.72) and was also seen when stigma was characterized as a continuous variable (see Table 4, Supplemental Digital Content, http://links.lww.com/QAI/B538). Those reporting internalized stigma also reported that they were less likely to have ever started ART or currently be taking ART and more likely to report having ever hidden pills and to have been recently nonadherent (see Table 5, Supplemental Digital Content, http://links.lww.com/QAI/B538).

There was little evidence of interaction between study arm, viral suppression, and any of the other stigma exposures (see Table 6, Supplemental Digital Content, http://links.lww.com/QAI/B538). In the exploratory analysis, the patterns were similar when stigma exposures were measured at PC0 or PC12, except that the association between internalized stigma and viral suppression became statistically weak (see Tables 7–12, Supplemental Digital Content, http://links.lww.com/QAI/B538).

At community level, there was one outlier community with a low viral suppression rate (29.3%), with the others ranging from 54.4% to 80.0% (Fig. 1). There was some, but limited, variation in the average level of stigma across communities. There was little evidence that the proportion of PC-HIV+ who were virally suppressed in a community was associated with any of the community-level measures of stigma as measured among PC-HIV+^SR^, PC-HIV−, HWs, or CHiPs (Fig. 1). These results were similar when community-level stigma measures from earlier rounds of data collection were used (see Figures 4 and 5, Supplemental Digital Content, http://links.lww.com/QAI/B538).

## DISCUSSION

At 24 months into the HPTN (071) PopART cluster-randomized trial in 21 study communities in Zambia and South Africa, 69.1% of the randomly selected research PC participants living with HIV were virally suppressed. Viral suppression was lowest among PLHIV who did not report that they were living with HIV in the research interview. Among self-identifying PLHIV, those who reported internalized stigma were less likely to be virally suppressed than those who did not, were also less likely to report ever or current ART use, and were more likely to report hiding pills or recent non-adherence to ART. Experienced stigma in the community or health setting and perceived stigma were not associated with poorer viral suppression. The associations were similar across all 3 arms of the trial under different approaches to the delivery of HIV testing and treatment. The viral suppression rate among PLHIV was not associated with any of the community-level measures of stigma reflected in the responses of PLHIV, community members, or HWs.

This was a large study of a representative random sample of PLHIV in 21 communities, with data on a valid, biologically measured, outcome variable (viral suppression) reflective of the cumulative success of efforts to support PLHIV along the continuum of care, from diagnosis, to linkage to care, to adherence to antiretroviral medication. We used theory-based, harmonized, and validated measures of a range of dimensions of stigma.^15^

Nevertheless, our study had limitations that may have relevance to future studies on this subject. First, although we had high response rates, we were missing data on stigma among an important group: PLHIV who did not self-report their HIV-positive status. Lack of self-report of a positive HIV status might be because HIV-positive status was not yet diagnosed (in which case participants would not be on ART and would also not have had a chance to experience HIV stigma) or because participants chose not to reveal their status to an interviewer (in which case they may in fact be on ART, but stigma may also be relevant to their decision not to disclose to the interviewer). The overall impact of these potentially different effects on the association of interest in this article is not clear. Second, despite many years of research into measurement of stigma and our use of best-practice measures, stigma remains a complex and evolving phenomenon, potentially subject to reporting biases. Therefore, the items we included to assess stigma may not have captured all the subtle experiences of stigma of PLHIV in our study setting.

Four other studies have explored measures of HIV stigma and their association with viral suppression.^18–21^ Among a cohort of 6448 PLHIV in care in the United States, 88% of whom were virally suppressed, a “modest but significant” association was observed between internalized stigma and concurrent viremia. This followed a smaller study in the United States nested within a randomized trial of an intervention to reduce HIV stigma among 234 African American women in HIV care in 3 cities, in which changes in viral load over time were associated with experiences of stigma.^19^ In another US study, among 92 young Black men who have sex with men, those who reported higher levels of total stigma and personalized stigma were less likely to be virally suppressed.^21^ These studies build on a wealth of evidence from multiple settings that suggest that stigma is a barrier to the continuum of care for PLHIV. Our finding of an association between current internalized stigma and viral suppression aligns with existing research, but it is unexpected in suggesting that experience of stigma, perception of stigma, and community-level stigma were not associated with viral suppression in our study communities.

Why might we have seen this unexpected finding? Previous reviews may have been more likely to report findings of an association than if they did not find one with each of the steps of the treatment cascade. Alternatively, the context of our study may differ. In 2 arms of the study, intensive, home-based interventions were being delivered to test the whole community and link and initiate antiretroviral treatment for all PLHIV, and this may explain the absence of an effect. Levels of knowledge of HIV status were high across all the study communities, especially in arms A and B. Furthermore, levels of viral suppression were lower among PLHIV who did not self-report their HIV status (and therefore did not answer questions on HIV stigma). Ongoing efforts to reach the undiagnosed fraction with testing remain essential. It is hard to unpick the importance of stigma as a barrier to reaching the undiagnosed. However, there is growing recognition of the importance of HIV transmission in early infection. Universal test and treat approaches based on annual testing cycles may struggle to address this. Newly infected individuals involved in concurrent partnerships, including highly stigmatized groups such as female sex workers and their clients, men who have sex with men, and transgender individuals, may be particularly vulnerable. Public health messaging that emphasizes the importance of frequent HIV testing, and testing after potential exposure to HIV, continues to be essential. Differentiated services, including programs that offer testing and prevention at a more frequent rate for stigmatized groups involved in such sexual networks will need to overcome stigma.

HIV programs must also work toward the elimination of HIV transmission during the period between an HIV diagnosis and viral suppression. Linkage to care was lower among those experiencing stigma in a universal test and treat trial in South Africa, and linkage to care was slower among those feeling shame about an HIV diagnosis in a case–control study nested within the trial reported in this article.^22^ Post-HIV test counseling, including with couples, is critical and must emphasize messages that overcome the impact of stigma on slow linkage to care. These include (1) that treatment can be sought rapidly to improve individual health, (2) that safe sex should be observed during the period between a diagnosis and initiating treatment and attaining a suppressed viral load, and, (3) counseling services and/or peer support that limits the development of internalized stigma, supports appropriate disclosure to foster social support, and strengthens resilience is critically important, and PLHIV should have access to these services.

Finally, stigma can reduce treatment adherence if participants experience mental health problems or are reluctant to be seen at clinic or identified as living with HIV.^6^ Previous research has identified a potentially strong effect of internalized stigma on mental health and treatment decisions and adherence.^23–26^ Our findings are in line with these studies. In our qualitative work, anticipation of stigma, linked to feelings of low worth, was an important component of narratives of people in these communities and their decisions to initiate or stay on treatment. In an earlier analysis, we found that SR ART adherence was lower among those who had experienced and internalized stigma among PLHIV who started treatment before the PopART trial and were enrolled at PC0.^27^

Health services must therefore continue to strive to minimize the effects of stigma on access to treatment. Although there was no association in our study between health service setting stigma and viral suppression at community level, the pathways between such experiences and health outcomes remain unclear. Our qualitative research highlights how critical friendly health services are for patients.^6^ HWs who are themselves living with HIV may have an important role. Clinical treatment services must play close attention to the mental health of patients on treatment. Although there is some evidence that the prevalence of HIV stigma in Africa is reducing as testing and treatment scale-up,^28–30^ efforts to reduce stigma must continue at multiple levels and have the potential to catalyze continued strengthening of HIV control efforts.

## Supplementary Material

SUPPLEMENTARY MATERIAL
