# Supplementary material for: HIV Stigma and Viral Suppression Among People Living With HIV in the Context of Universal Test and Treat: Analysis of Data From the HPTN 071 (PopART) Trial in Zambia and South Africa
Source: J Acquir Immune Defic Syndr. 2020 Sep 24;85(5):561–70. doi: 10.1097/QAI.0000000000002504 (PMC7654947; doi:10.1097/QAI.0000000000002504)
Supplement: SUPPLEMENTARY MATERIAL [file qai-85-561-s001.docx]

**Supplementary materials**

**
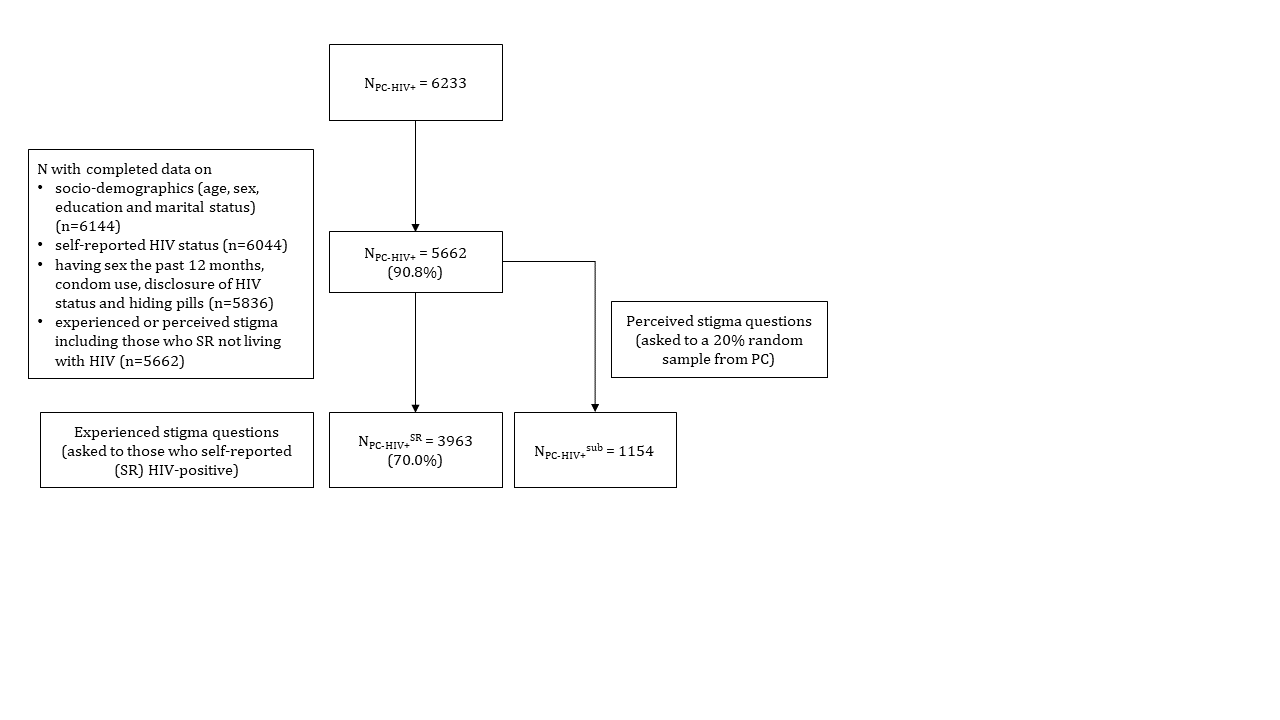
**

**Figure S1.** Flowchart with (a) the PC-HIV+ population (n=5662), (b) the PC-HIV+^SR^ who received the questions on experienced stigma (n=3963) and (c) the 20% random sample from the population cohort (PC) who received questions on perceived stigma (PC-HIV+^sub^, n=1154).

**
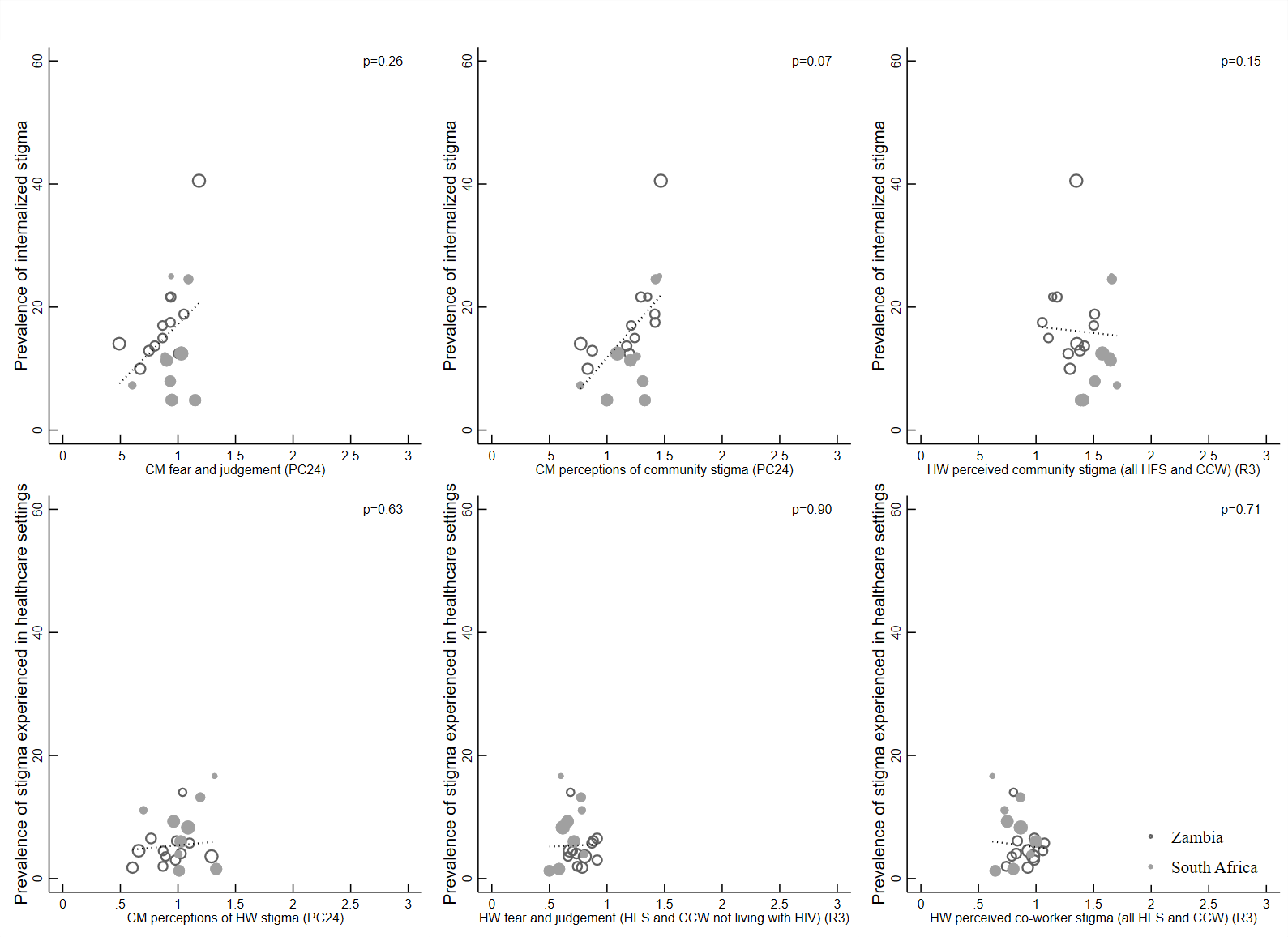
**

**Figure S2.** Linear regression analysis looking at the association between community level internalised stigma and stigma experienced in healthcare settings with fear and judgement and perceived stigma reported by community members and healthcare workers at PC24 and R3.

Each circle represents one cluster. Size of the circles are proportional to the number of PLHIV respondents in each cluster. Dotted lines reflect linear regression slopes from cluster-level analyses of the associations and weighted by the size of the PLHIV community in each cluster.

**
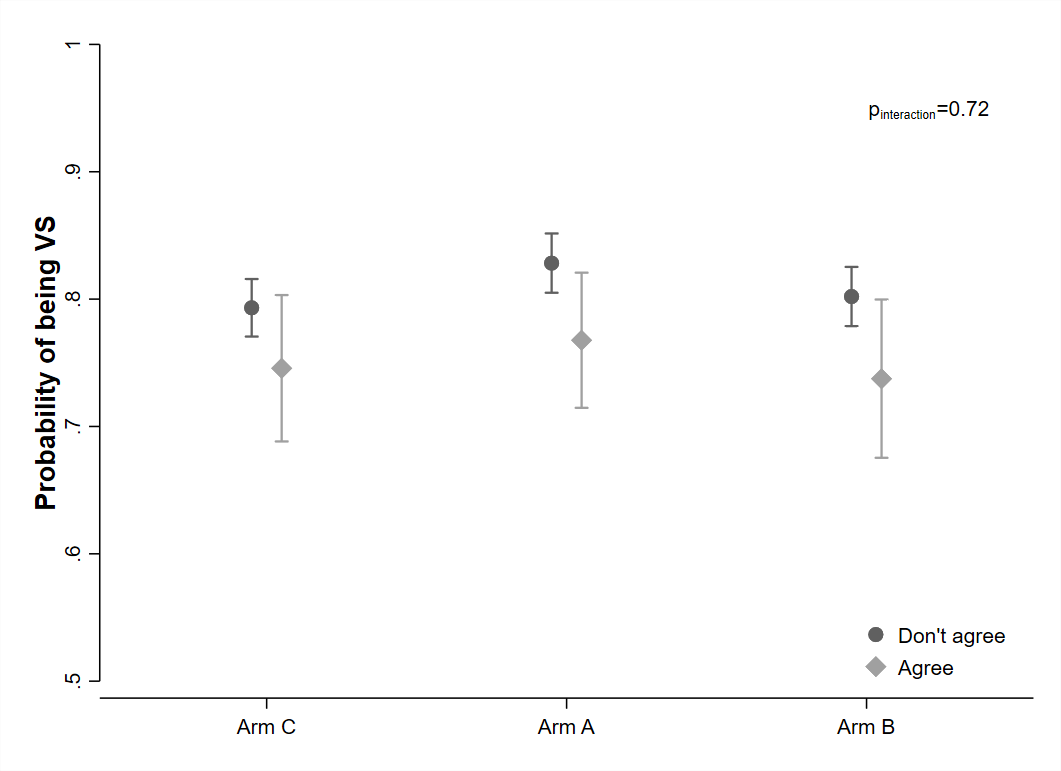
**

**Figure S3.** Levels of viral suppression by internalized stigma and study arm among 3963 PC-HIV+^SR^.

**
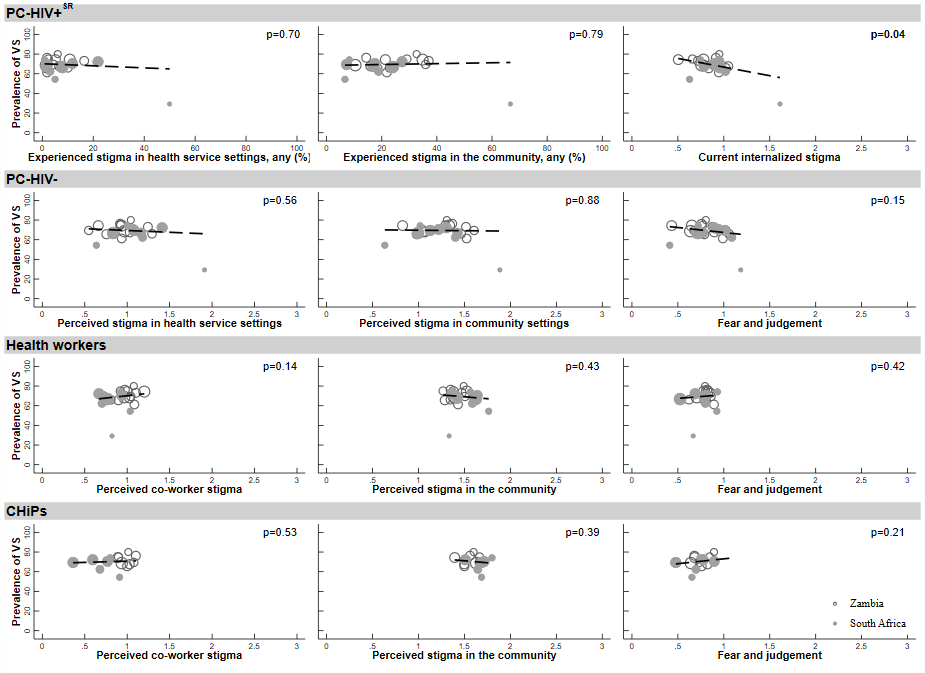
**

**Figure S4.** The association between the beliefs and perceptions of PLHIV, community members and health workers at baseline (PC0 and R1) and levels of viral suppression at PC24 among 6233 lab confirmed HIV positive participants in 21 communities in South Africa and Zambia.

**
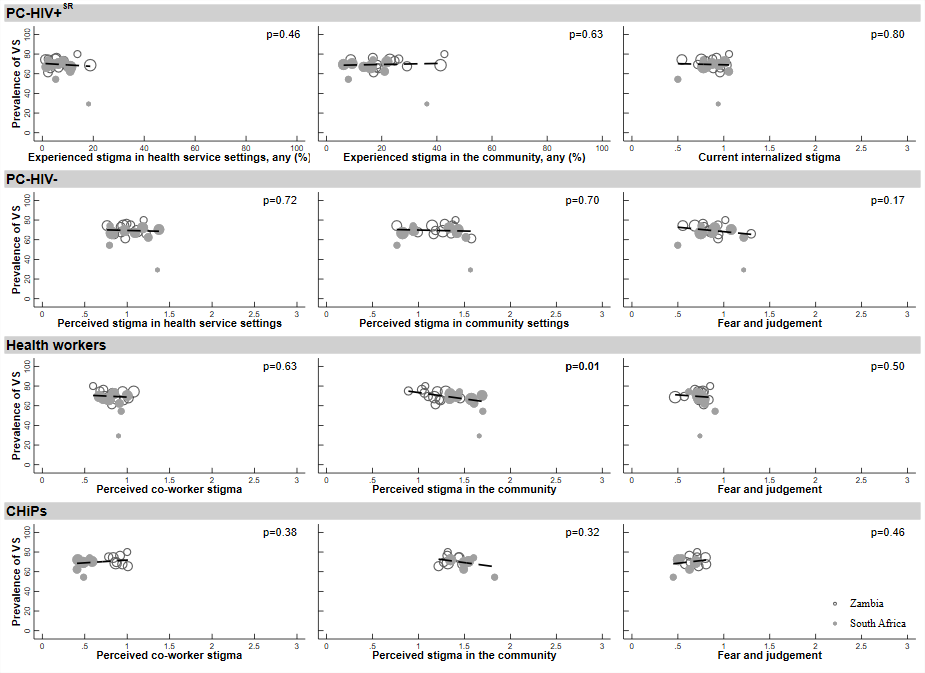
**

**Figure S5.** The association between the beliefs and perceptions of PLHIV, community members and health workers at PC12 and R2 (at 12 months) and levels of viral suppression at PC24 among 6233 lab confirmed HIV positive participants in 21 communities in South Africa and Zambia.

**Table S1.** Differences between PC-HIV+ that were excluded and those included in the main analysis.

|  | **Excluded**  **(n=571)** | | **Included**  **(n=5662)** | | **P value (x^2^)** |
| --- | --- | --- | --- | --- | --- |
|  | **N** | **%** | **N** | **%** |  |
| **Country** |  |  |  |  | **0.017 (5.66)** |
| Zambia | 323 | 8.5 | 3491 | 91.5 |  |
| South Africa | 248 | 10.3 | 2171 | 89.7 |  |
| **Study arm** |  |  |  |  | **<0.001 (29.07)** |
| Arm A | 256 | 11.9 | 1903 | 88.1 |  |
| Arm B | 142 | 7.5 | 1749 | 92.5 |  |
| Arm C | 173 | 7.9 | 2010 | 92.1 |  |
| **Sex** |  |  |  |  | 0.750 (0.10) |
| Male | 77 | 8.9 | 791 | 91.1 |  |
| Female | 494 | 9.2 | 4871 | 90.8 |  |
| **Age groups** |  |  |  |  | 0.128 (4.12) |
| 18-24 | 93 | 7.8 | 1105 | 92.2 |  |
| 25-34 | 274 | 9.8 | 2527 | 90.2 |  |
| 35-44 | 204 | 9.1 | 2030 | 90.9 |  |
| Total | 571 | 9.2 | 5662 | 90.8 |  |
| **Viral suppression status** |  |  |  |  | 0.064 (3.43) |
| Not suppressed | 155 | 8.1 | 1749 | 91.9 |  |
| Suppressed | 416 | 9.6 | 3913 | 90.4 |  |

| **Table S2.** Description of stigma exposure variables | |  | |  |  |  |  |
| --- | --- | --- | --- | --- | --- | --- | --- |
| **Population group and exposure** | **Stigma items / statements** | **Treated in analysis** |  | **Cronbach’s alpha (items)** | | | |
|  |  |  |  | PC0/R1 | PC12/R2 | PC24/R3 | PC24 (n=5662) |
| **PC-HIV+^SR^ (Four individual-level and three community-level exposures)** | | |  |  |  |  |  |
| *Any reported internalized stigma (Three items)* | - I have lost respect or standing in the community because of my HIV status - I think less of myself because of my HIV status - I have felt ashamed because of my HIV status | *Individual-level analysis*  4-item Likert scale (Strongly agree to strongly disagree). We collapsed responses to each statement to create one binary variable (i.e. “agree “ versus “disagree“). We combined the three variables into one composite binary variable describing whether PLHIV agreed to feeling any of the three manifestations of current internalized stigma (“agree“ vs “disagree“ to any of the three statements)  We calculated a continuous stigma score by taking the average of the three items using the individual-level scores (possible values from 0 to 3)  *Community-level analysis*  We calculated community-level scores by taking the average of the three items using the individual-level scores (scores 0 to 3) and then collapsing these to the community level. This resulted in average community-level scores with a theoretical range from 0 (all answers of all individuals ‘Strongly Disagree’) to 3 (all answers of all individuals ‘Strongly Agree’) | | 0.92 (5) | 0.89 (5) | 0.86 (5) | 0.84 (5) |
| *Any reported experienced stigma in the community (Five items)* | - People have talked badly about me because of my HIV status - Someone else disclosed my HIV status without my permission - I have been verbally insulted, harassed and/or threatened because of my HIV status - I have been physically assaulted because of my HIV status - I have felt that people have not wanted to sit next to me, for example, on public transport, at church or in a waiting room because of my HIV status | *Individual-level and community-level analysis*  Frequency of experiences in the last 12 months (Never, once, a few times, often). We collapsed responses to each statement to create one binary variable (i.e. “never“ versus “at least once“). We combined the five variables into one composite binary variable capturing any experience of stigma in the community (“never“ versus “at least once“ to any of the five statements) | | 0.90 (3) | 0.83 (3) | 0.82 (3) | 0.79 (3) |
| *Any reported experienced stigma in health service settings (Three items)* | - I have been denied health services because of my HIV status - Healthcare workers talked badly about me because of my HIV status - A health worker disclosed my HIV status without my permission | *Individual-level and community-level analysis* Frequency of experiences in the last 12 months (Never, once, a few times, often). We collapsed responses to each statement to create one binary variable (i.e. “never“ versus “at least once“). We combined the three variables into one composite binary variable capturing any experience of stigma in healthcare settings (“never“ versus “at least once“ to any of the five statements) | | 0.77 (2) | 0.68 (2) | 0.72 (2) | 0.74 (2) |
| *Any stigma (Eleven items)* | - Items above | *Individual-level analysis only*  As above | | 0.87 (11) | 0.87 (11) | 0.86 (11) | 0.82 (11) |
| **PC-HIV- and PC-HIV+ (Two individual-level and three community-level exposures)** | | | |  |  |  |  |
| *Any negative attitudes (Fear and judgment using three items)*  **PC-HIV- only** | - I fear that I could contract HIV if I come into contact with the saliva of a person living with HIV - I would not like to sit close to someone living with HIV, for example on public transport, at church or in a waiting room - I would be ashamed if someone in my family had HIV | *Community-level analysis only*  We calculated community-level scores by taking the average of the three items using the individual-level scores (scores 0 to 3) and then collapsing these to the community level. This resulted in average community-level scores with a theoretical range from 0 (all answers of all individuals ‘Strongly Disagree’) to 3 (all answers of all individuals ‘Strongly Agree’) | | 0.72 (3) | 0.76 (3) | 0.80 (3) |  |
| *Any perceived stigma in community settings (Five items)* | - People thought to be living with HIV are sometimes physically assaulted - People sometimes talk badly about PLHIV to others - People thought to be living with HIV lose respect or standing - People thought to be living with HIV are verbally insulted, harassed and/or threatened - People sometimes disclose that other people are HIV positive without their permission | *Individual-level analysis*  4-item Likert scale (Strongly agree to strongly disagree). We collapsed responses to each statement to create one binary variable (i.e. “agree“ versus “disagree “). We combined the five variables into one composite binary variable capturing any perceived stigma in the community (“agree“ versus “disagree“ to any of the five statements).  We calculated a continuous stigma score by taking the average of the five items using the individual-level scores (possible values from 0 to 3)  *Community-level analysis*  We calculated community-level scores by taking the average of the five items using the individual-level scores (scores 0 to 3) and then collapsing these to the community level. This resulted in average community-level scores with a theoretical range from 0 (all answers of all individuals ‘Strongly Disagree’) to 3 (all answers of all individuals ‘Strongly Agree’) | | 0.84 (5) | 0.84 (5) | 0.86 (5) | 0.85 (5) |
| *Any perceived stigma in health service settings (Two items)* | - Health workers sometimes talk badly about people living with or thought to be living with HIV to others - Health workers sometimes disclose that other people are HIV positive without their permission | *Individual-level analysis*  4-item Likert scale (Strongly agree to strongly disagree). We collapsed responses to each statement to create one binary variable (i.e. “agree“ versus “disagree “). We combined the two variables into one composite binary variable capturing any perceived stigma healthcare settings (“agree“ versus “disagree“ to any of the five statements).  We calculated a continuous stigma score by taking the average of the two items using the individual-level scores (possible values from 0 to 3)  *Community-level analysis*  We calculated community-level scores by taking the average of the two items using the individual-level scores (scores 0 to 3) and then collapsing these to the community level. This resulted in average community-level scores with a theoretical range from 0 (all answers of all individuals ‘Strongly Disagree’) to 3 (all answers of all individuals ‘Strongly Agree’) | | 0.77 (2) | 0.68 (2) | 0.72 (2) | 0.74 (2) |
| **HW and CHiPs (Three exposures)** | | |  |  |  |  |  |
| *Any negative attitudes (Fear and judgment using five items)*  **HW-HIV- only** | - I fear that I could contract HIV if I come into contact with the saliva of a person living with HIV - I avoid physical contact with clients living with HIV - HIV is punishment from God - Other people deserve access to health services more than PLHIV - I would be ashamed if someone in my family had HIV | *Community-level analysis*  4-item Likert scale (Strongly agree to strongly disagree). We calculated community-level scores by taking the average of the five items using the individual-level scores (scores 0 to 3) and then collapsing these to the community level. This resulted in average community-level scores with a theoretical range from 0 (all answers of all individuals ‘Strongly Disagree’) to 3 (all answers of all individuals ‘Strongly Agree’) | | **CHiPs** | | |  |
|  |  |  |  | 0.50 (5) | 0.68 (5) | 0.64 (5) |  |
|  |  |  |  | **HW** | | |  |
|  |  |  |  | 0.67 (5) | 0.66 (5) | 0.66 (5) |  |
| *Any perceived stigma in the community (Five items)* | - People thought to be living with HIV are sometimes physically assaulted - People sometimes talk badly about PLHIV to others - People thought to be living with HIV lose respect or standing - People thought to be living with HIV are verbally insulted, harassed, or threatened - People hesitate to start ARV drugs because they are afraid others will learn they are living with HIV | *Community-level analysis*  4-item Likert scale (Strongly agree to strongly disagree). We calculated community-level scores by taking the average of the four items using the individual-level scores (scores 0 to 3) and then collapsing these to the community level. This resulted in average community-level scores with a theoretical range from 0 (all answers of all individuals ‘Strongly Disagree’) to 3 (all answers of all individuals ‘Strongly Agree’) | | **CHiPs** | | |  |
|  |  |  |  | 0.61 (5) | 0.66 (5) | 0.69 (5) |  |
|  |  |  |  | **HW** | | |  |
|  |  |  |  | 0.67 (5) | 0.78 (5) | 0.73 (5) |  |
| *Any perceived co-worker stigma (Four items)* | - My co-workers sometimes talk badly about people thought to be living with HIV - My co-workers sometimes gossip about clients' HIV test results - My co-workers sometimes treat people living with HIV poorly when providing them with health services - My co-workers sometimes verbally insult clients living with HIV | *Community-level analysis*  4-item Likert scale (Strongly agree to strongly disagree). We calculated community-level scores by taking the average of the four items using the individual-level scores (scores 0 to 3) and then collapsing these to the community level. This resulted in average community-level scores with a theoretical range from 0 (all answers of all individuals ‘Strongly Disagree’) to 3 (all answers of all individuals ‘Strongly Agree’) | | **CHiPs** | | |  |
|  |  |  |  | 0.78 (4) | 0.76 (4) | 0.78 (4) |  |
|  |  |  |  | **HW** | | |  |
|  |  |  |  | 0.77 (4) | 0.82 (4) | 0.78 (4) |  |

CHiPs: Community HIV care Providers; HW: Facility-based and community based health workers; HW-HIV-: HW or CHiPs who self reported not living with HIV; PC: Population cohort; PC-SR-HIV+: Participant in the population cohort who self-reported living with HIV; R: Round.

| **Table S3.** The association between internalised stigma with stigma experienced in the community and in healthcare settings at PC24 in 21 communities in Zambia and South Africa (n=3963) | | | | | |
| --- | --- | --- | --- | --- | --- |
| **Variable** | | **Current internalized stigma** | | **Stigma experienced in the community** | |
|  |  | **aOR ^a^ (95% CI)** | **aOR ^b^ (95% CI)** | **aOR ^a^ (95% CI)** | **aOR ^b^ (95% CI)** |
| Stigma experienced in health service settings | Never | 1.00 | 1.00 | 1.00 | 1.00 |
|  | At least once | **3.98 (2.88-5.51)** | **4.05 (2.92-5.62)** | **20.31 (13.87-29.75)** | **20.33 (13.89-29.77)** |
| Stigma experienced in the community | Never | 1.00 | 1.00 | - | - |
|  | At least once | **4.26 (3.49-5.20)** | **4.30 (3.52-5.25)** | - | - |

aOR, adjusted odds ratio using logistic regression; CI, confidence interval. Bolded, p<0.05.

^a^ Model adjusted for age group, sex, and community with robust standard errors.

^b^ Model adjusted for age group, sex, education, marital status, time of diagnosis and community with robust standard errors.

| **Table S4.** The association between internalised and perceived HIV stigma and viral suppression at PC24 in 21 communities in Zambia and South Africa | | |
| --- | --- | --- |
| **Variable** | **aRR ^a^ (95% CI)** | **aRR ^b^ (95% CI)** |
| Current internalized stigma (n=3963) | **0.97 (0.95-1.00)** | 0.97 (0.95-1.00) |
| Perceived stigma in health service settings (n=1154) ^c^ | 1.03 (0.96-1.09) | 1.02 (0.96-1.09) |
| Perceived stigma in the community (n=1154) ^c^ | 1.03 (0.97-1.10) | 1.02 (0.95-1.08) |

aRR, adjusted risk ratio using ‘modified’ Poisson regression; CI, confidence interval. Bolded, p<0.05. Internalized and perceived stigma treated as continuous variable (possible score from 0 to 3, higher value indicates more stigma).

^a^ Model adjusted for age group, sex, and community with robust standard errors.

^b^ Model adjusted for age group, sex, education, marital status, time of diagnosis and community with robust standard errors.

^c^ Stigma items captured by PLHIV who completed the extended questionnaire (random 20% sample of the Population Cohort).

| **Table S5.** The association between internalised stigma, ever and currently taking ART, hiding pills and ART non-adherence at PC24 in 21 communities in Zambia and South Africa (n=3963) | | | | | | | | | |
| --- | --- | --- | --- | --- | --- | --- | --- | --- | --- |
| **Variable** | | **Currently on ART (n=3,963)** | | **Ever on ART**  **(n=3,963)** | | **Hiding pills**  **(n=3,346)** | | **ART non-adherence**  **(n=3,341)** | |
|  |  | **aOR ^a^**  **(95% CI)** | **aOR ^b^**  **(95% CI)** | **aOR ^a^**  **(95% CI)** | **aOR ^b^**  **(95% CI)** | **aOR ^a^**  **(95% CI)** | **aOR ^b^**  **(95% CI)** | **aOR ^a^**  **(95% CI)** | **aOR ^b^**  **(95% CI)** |
| Internalised stigma | Don’t agree | 1.00 | 1.00 | 1.00 | 1.00 | 1.00 | 1.00 | 1.00 | 1.00 |
|  | Agree | **0.60**  **(0.48-0.75)** | **0.61**  **(0.48-0.76)** | **0.67**  **(0.53-0.84)** | **0.68**  **(0.53-0.86)** | **1.36**  **(1.06-1.74)** | **1.38**  **(1.07-1.77)** | **1.69**  **(1.30-2.21)** | **1.70**  **(1.30-2.23)** |
| Internalised stigma ^c^ |  | **0.85**  **(0.73-0.98)** | **0.85**  **(0.73-1.00)** | 0.91  (0.78-1.07) | 0.93  (0.79-1.09) | **1.18**  **(1.01-1.37)** | **1.18**  **(1.02-1.38)** | **1.40**  **(1.17-1.67)** | **1.40**  **(1.17-1.67)** |

aOR, adjusted odds ratio using logistic regression; CI, confidence interval. Bolded, p<0.05.

^a^ Model adjusted for age group, sex, and community with robust standard errors.

^b^ Model adjusted for age group, sex, education, marital status, time of diagnosis and community with robust standard errors.

^c^ Internalized stigma treated as continuous variable (possible score from 0 to 3, higher value indicates more stigma).

| **Table S6.** Statistical interaction of stigma variables, disclosure of HIV status and hiding ART pills by arm on viral suppression. | | | |
| --- | --- | --- | --- |
| **Stigma and other exposures** | **P value for interaction** | | |
|  | **PC0** | **PC12** | **PC24** |
| Any stigma | 0.29 | 0.24 | 0.11 |
| Experienced stigma in health service settings | 0.41 | 0.41 | 0.23 |
| Experienced stigma in the community | 0.97 | 0.13 | 0.34 |
| Current internalized stigma | 0.07 | 0.31 | 0.72 |
| Disclosed HIV status | 0.30 | 0.72 | 0.37 |
| Have you ever hidden your ART pills? | 0.22 | 0.19 | 0.17 |
| Perceived stigma in the community | 0.21 | 0.89 | 0.25 |
| Perceived stigma in health service settings | **0.03** | 0.65 | 0.25 |

All models adjusted for age group, sex, education, marital status, time of diagnosis and triplet with robust standard errors. P values presented for the following interaction terms: arm by stigma exposure variables, arm by disclosed HIV status, and arm by hiding ART pills.

| **Table S7.** Characteristics of PC-HIV+ at PC0 with viral suppression data at PC24 in 21 communities in Zambia and South Africa (n=3841^a^). | | | | | | | |
| --- | --- | --- | --- | --- | --- | --- | --- |
|  |  | **Zambia** | | **South Africa** | | **Total** | |
|  |  | **N** | **%** | **N** | **%** | **N** | **%** |
| ***Sociodemographics*** | ***Categories*** |  |  |  |  |  |  |
| Sex | Male | 315 | 14.6 | 218 | 12.9 | 533 | 13.9 |
|  | Female | 1837 | 85.4 | 1471 | 87.1 | 3308 | 86.1 |
| Age groups | 18-24 | 419 | 19.5 | 270 | 16.0 | 689 | 17.9 |
|  | 25-34 | 914 | 42.5 | 783 | 46.4 | 1697 | 44.2 |
|  | 35-44 | 819 | 38.1 | 636 | 37.7 | 1455 | 37.9 |
| Education groups | Did not complete secondary | 981 | 45.6 | 256 | 15.2 | 1237 | 32.2 |
|  | Completed secondary | 1075 | 50.0 | 1378 | 81.6 | 2453 | 63.9 |
|  | Further | 96 | 4.5 | 55 | 3.3 | 151 | 3.9 |
| Marital status | Married | 1257 | 58.4 | 450 | 26.6 | 1707 | 44.4 |
|  | Never married | 317 | 14.7 | 1157 | 68.5 | 1474 | 38.4 |
|  | Divorced/separated | 380 | 17.7 | 60 | 3.6 | 440 | 11.5 |
|  | Widowed | 198 | 9.2 | 22 | 1.3 | 220 | 5.7 |
| Study arm | Arm A | 660 | 30.7 | 507 | 30.0 | 1167 | 30.4 |
|  | Arm B | 766 | 35.6 | 600 | 35.5 | 1366 | 35.6 |
|  | Arm C | 726 | 33.7 | 582 | 34.5 | 1308 | 34.1 |
| ***Test and treatment status*** |  |  |  |  |  |  |  |
| Viral suppression status at PC24 | Not suppressed | 609 | 28.3 | 556 | 32.9 | 1165 | 30.3 |
|  | Suppressed | 1543 | 71.7 | 1133 | 67.1 | 2676 | 69.7 |
| Self-Reported HIV testing and treatment status | Last test HIV-positive on ART | 895 | 41.6 | 560 | 33.2 | 1455 | 37.9 |
|  | Last test HIV-positive, but not currently on ART or with no data on ART | 360 | 16.7 | 229 | 13.6 | 589 | 15.3 |
|  | Last test HIV-negative | 469 | 21.8 | 385 | 22.8 | 854 | 22.2 |
|  | Don't know HIV status | 278 | 12.9 | 378 | 22.4 | 656 | 17.1 |
|  | Never tested | 150 | 7.0 | 137 | 8.1 | 287 | 7.5 |
| ART adherence (n=1467) ^b^ | Yes | 758 | 84.6 | 501 | 87.7 | 1259 | 85.8 |
|  | No (poor adherence) | 138 | 15.4 | 70 | 12.3 | 208 | 14.2 |
| Time of diagnosis ^c^ | Before PopART | 947 | 44.0 | 658 | 39.0 | 1605 | 41.8 |
|  | During PopART | 212 | 9.9 | 63 | 3.7 | 275 | 7.2 |
|  | SR HIV+ with missing dates | 96 | 4.5 | 68 | 4.0 | 164 | 4.3 |
|  | Did not self-report HIV positive | 897 | 41.7 | 900 | 53.3 | 1797 | 46.8 |
| ***HIV stigma*** |  |  |  |  |  |  |  |
| Any stigma ^d^ | No stigma experienced | 759 | 60.5 | 544 | 68.9 | 1303 | 63.7 |
|  | Any stigma experienced | 496 | 39.5 | 245 | 31.1 | 741 | 36.3 |
|  | Did not self-report HIV positive | 897 |  | 900 |  | 1797 |  |
| Experienced stigma in health service settings (n=2044) ^d^ | Never | 1169 | 93.1 | 707 | 89.6 | 1876 | 91.8 |
|  | At least once | 86 | 6.9 | 82 | 10.4 | 168 | 8.2 |
| Experienced stigma in the community (n=2044) ^d^ | Never | 946 | 75.4 | 625 | 79.2 | 1571 | 76.9 |
|  | At least once | 309 | 24.6 | 164 | 20.8 | 473 | 23.1 |
| Current internalized stigma (n=2044) ^d^ | Don't agree | 951 | 75.8 | 641 | 81.2 | 1592 | 77.9 |
|  | Agree | 304 | 24.2 | 148 | 18.8 | 452 | 22.1 |
| Perceived stigma in health service settings (n=720) ^e^ | No | 296 | 69.6 | 198 | 67.1 | 494 | 68.6 |
|  | Yes | 129 | 30.4 | 97 | 32.9 | 226 | 31.4 |
| Perceived stigma in the community (n=720) ^e^ | No | 115 | 27.1 | 143 | 48.5 | 258 | 35.8 |
|  | Yes | 310 | 72.9 | 152 | 51.5 | 462 | 64.2 |
| Disclosed HIV status | No | 100 | 8.0 | 50 | 6.3 | 150 | 7.3 |
|  | Yes | 1155 | 92.0 | 739 | 93.7 | 1894 | 92.7 |
|  | Did not self-report HIV positive | 897 |  | 900 |  | 1797 |  |
| Have you ever hidden your ART pills? ^f^ | No | 569 | 63.5 | 510 | 89.2 | 1079 | 73.5 |
|  | Yes | 327 | 36.5 | 62 | 10.8 | 389 | 26.5 |
|  | Did not self-report HIV positive or ever start ART | 1256 |  | 1117 |  | 2373 |  |

^a^ 3841 of 4158 (92.4%) with complete data on viral suppression at PC24 and marital status, self-reported HIV status, experienced or perceived stigma, disclosure of HIV status and hiding pills at PC0.

^b^ Poor or non-adherence was defined as ‘respondents self-reporting that they had ever started ART but were not currently taking ART, or currently taking ART but had either stopped in the past 12 months, or missed pills in the past seven days’.

^c^ Time of first positive HIV test result was asked only to those who self-reported HIV-positive.

^d^ Stigma items captured by PC-HIV+^SR^.

^e^ Stigma items captured by PC-HIV+^sub^ who completed the extended questionnaire (random 20% sample of the Population Cohort.

^f^ Asked to everyone but those ever started on ART.

| **Table S8.** The association between sociodemographic characteristics, ART adherence and self-reported HIV status at PC0 with viral suppression at PC24 in 21 communities in Zambia and South Africa (n=3841) | | | |
| --- | --- | --- | --- |
| **Variable** | **Categories** | ***% virally suppressed***  ***n*/*N* (%)** | **aRR ^a^ (95% CI)** |
| Country ^b^ | Zambia | 1543/2152 (71.7%) | 1.00 |
|  | South Africa | 1133/1689 (67.1%) | **0.92 (0.88-0.96)** |
| Age group ^c^ | 18-24 | 331/689 (48.0%) | 1.00 |
|  | 25-34 | 1193/1697 (70.3%) | **1.47 (1.35-1.59)** |
|  | 35-44 | 1152/1455 (79.2%) | **1.66 (1.53-1.80)** |
| Sex | Male | 322/533 (60.4%) | 1.00 |
|  | Female | 2354/3308 (71.2%) | **1.20 (1.12-1.29)** |
| ART adherence (n=1467) ^d^ | Yes | 1117/1259 (88.7%) | 1.00 |
|  | No (poor adherence) | 173/208 (83.2%) | **0.92 (0.87-0.98)** |
| Self-Reported HIV status ^e^ | Last test HIV-positive on ART | 1286/1455 (88.4%) | 1.00 |
|  | Last test HIV-positive, but not currently on ART or with no data on ART | 414/589 (70.3%) | **0.80 (0.76-0.85)** |
|  | Last test HIV-negative | 448/854 (52.5%) | **0.64 (0.60-0.69)** |
|  | Don't know HIV status | 387/656 (59.0%) | **0.70 (0.66-0.75)** |
|  | Never tested | 141/287 (49.1%) | **0.61 (0.55-0.69)** |

aRR, adjusted risk ratio; CI, confidence interval; n, number of individuals that are virally suppressed; N, total number of individuals within groups; SR: Self-reported. Bolded, p<0.05.

^a^ Adjusted for age group, sex and community with robust standard errors.

^b^ The aRR is adjusted for age group and sex with robust standard errors.

^c^ Overall p value for age group: p<0.001.

^d^ Poor or non-adherence was defined as ‘respondents self-reporting that they had ever started ART but were not currently taking ART, or currently taking ART but had either stopped in the past 12 months, or missed pills in the past seven days’.

^e^ Overall p value for self-reported HIV testing and treatment status: p<0.001.

| **Table S9.** The association between experienced and perceived HIV stigma at PC0 and viral suppression at PC24 in 21 communities in Zambia and South Africa. | | | | |
| --- | --- | --- | --- | --- |
| **Variable** | **Categories** | ***n*/*N* (%)** | **aRR ^a^ (95% CI)** | **aRR ^b^ (95% CI)** |
| Self-report HIV status (n=3841) | Self-reported HIV-positive | 1700/2044 (83.2%) | 1.00 | 1.00 |
|  | Did not self-report HIV-positive | 976/1797 (54.3%) | **0.71 (0.67-0.74)** | **0.71 (0.68-0.75)** ^c^ |
| Any stigma (n=2044) | No stigma experienced | 1080/1303 (82.9%) | 1.00 | 1.00 |
|  | Any stigma experienced | 620/741 (83.7%) | 0.99 (0.95-1.03) | 1.00 (0.96-1.04) |
| Experienced stigma in health service settings (n=2044) | Never | 1554/1876 (82.8%) | 1.00 | 1.00 |
|  | At least once | 146/168 (86.9%) | 1.03 (0.97-1.10) | 1.03 (0.97-1.10) |
| Experienced stigma in the community (n=2044) | Never | 1304/1571 (83.0%) | 1.00 | 1.00 |
|  | At least once | 396/473 (83.7%) | 0.99 (0.94-1.04) | 0.99 (0.95-1.04) |
| Current internalized stigma (n=2044) | Disagree | 1326/1592 (83.3%) | 1.00 | 1.00 |
|  | Agree | 374/452 (82.7%) | 0.99 (0.94-1.03) | 0.99 (0.94-1.04) |
| Disclosed HIV status (n=2044) | No | 108/150 (72.0%) | 1.00 | 1.00 |
|  | Yes | 1592/1894 (84.1%) | **1.15 (1.04-1.28)** | **1.15 (1.04-1.28)** |
|  | Did not self-report HIV-positive | 976/1797 (54.3%) | -- | -- |
| Have you ever hidden your ART pills? (n=2044) | No | 944/1079 (87.5%) | 1.00 | 1.00 |
|  | Yes | 347/389 (89.2%) | 0.98 (0.94-1.03) | 0.98 (0.94-1.03) |
|  | Self-report HIV-positive but never started ART | 409/576 (71.0%) | -- | -- |
| Population group receiving the extended stigma questionnaire (n=3841) | Not receiving the perceived stigma questions | 2155/3121 (69.0%) | 1.00 | 1.00 |
|  | Random 20% sample of the PC | 521/720 (72.4%) | 1.04 (0.99-1.10) | 1.04 (0.99-1.09) |
| Perceived stigma in health service settings (n=720) ^d^ | No | 364/494 (73.7%) | 1.00 | 1.00 |
|  | Yes | 157/226 (69.5%) | 0.91 (0.82-1.02) | 0.92 (0.83-1.03) |
| Perceived stigma in the community (n=720) ^d^ | No | 186/258 (72.1%) | 1.00 | 1.00 |
|  | Yes | 335/462 (72.5%) | 0.98 (0.88-1.08) | 0.97 (0.87-1.07) |

aRR, adjusted risk ratio; CI, confidence interval; n, number of individuals that are virally suppressed; N, total number of individuals within groups. Bolded, p<0.05.

^a^ Model adjusted for age group, sex, and community with robust standard errors.

^b^ Model adjusted for age group, sex, education, marital status, time of diagnosis and community with robust standard errors.

^c^ Model adjusted for age group, sex, education, marital status and community with robust standard errors.

^d^ Stigma items captured by PLHIV who completed the extended questionnaire (random 20% sample of the Population Cohort).

| **Table S10.** Characteristics of PC-HIV+ at PC12 with viral suppression data at PC24 in 21 communities in Zambia and South Africa (n=4006^a^). | | | | | | | |
| --- | --- | --- | --- | --- | --- | --- | --- |
|  | | **Zambia** | | **South Africa** | | **Total** | |
|  | | **N** | **%** | **N** | **%** | **N** | **%** |
| ***Sociodemographics*** |  |  |  |  |  |  |  |
| Sex | Male | 329 | 13.2 | 199 | 13.1 | 528 | 13.2 |
|  | Female | 2160 | 86.8 | 1318 | 86.9 | 3478 | 86.8 |
| Age groups | 18-24 | 485 | 19.5 | 238 | 15.7 | 723 | 18.0 |
|  | 25-34 | 1063 | 42.7 | 702 | 46.3 | 1765 | 44.1 |
|  | 35-44 | 941 | 37.8 | 577 | 38.0 | 1518 | 37.9 |
| Education groups | Did not complete secondary | 1092 | 43.9 | 235 | 15.5 | 1327 | 33.1 |
|  | Completed secondary | 1270 | 51.0 | 1236 | 81.5 | 2506 | 62.6 |
|  | Further | 127 | 5.1 | 46 | 3.0 | 173 | 4.3 |
| Marital status | Married | 1497 | 60.1 | 474 | 31.2 | 1971 | 49.2 |
|  | Never married | 340 | 13.7 | 977 | 64.4 | 1317 | 32.9 |
|  | Divorced/separated | 430 | 17.3 | 53 | 3.5 | 483 | 12.1 |
|  | Widowed | 222 | 8.9 | 13 | 0.9 | 235 | 5.9 |
| Study arm | Arm A | 788 | 31.7 | 432 | 28.5 | 1220 | 30.5 |
|  | Arm B | 892 | 35.8 | 554 | 36.5 | 1446 | 36.1 |
|  | Arm C | 809 | 32.5 | 531 | 35.0 | 1340 | 33.4 |
| ***Test and treatment status*** |  |  |  |  |  |  |  |
| Viral suppression status at PC24 | Not suppressed | 677 | 27.2 | 492 | 32.4 | 1169 | 29.2 |
|  | Suppressed | 1812 | 72.8 | 1025 | 67.6 | 2837 | 70.8 |
| Self-Reported HIV testing and treatment status | Last test HIV-positive on ART | 1211 | 48.7 | 635 | 41.9 | 1846 | 46.1 |
|  | Last test HIV-positive, but not currently on ART or with no data on ART | 270 | 10.8 | 159 | 10.5 | 429 | 10.7 |
|  | Last test HIV-negative | 430 | 17.3 | 358 | 23.6 | 788 | 19.7 |
|  | Don't know HIV status | 244 | 9.8 | 129 | 8.5 | 373 | 9.3 |
|  | Never tested | 334 | 13.4 | 236 | 15.6 | 570 | 14.2 |
| ART adherence (n=1853) ^b^ | Yes | 1051 | 86.7 | 532 | 83.0 | 1583 | 85.4 |
|  | No (poor adherence) | 161 | 13.3 | 109 | 17.0 | 270 | 14.6 |
| Time of diagnosis ^c^ | Before PopART | 870 | 35.0 | 556 | 36.7 | 1426 | 35.6 |
|  | During PopART | 435 | 17.5 | 145 | 9.6 | 580 | 14.5 |
|  | SR HIV+ with missing dates | 176 | 7.1 | 93 | 6.1 | 269 | 6.7 |
|  | Did not self-report HIV positive | 1008 | 40.5 | 723 | 47.7 | 1731 | 43.2 |
| ***HIV stigma*** |  |  |  |  |  |  |  |
| Any stigma ^d^ | No stigma experienced | 928 | 62.7 | 619 | 78.0 | 1547 | 68.0 |
|  | Any stigma experienced | 553 | 37.3 | 175 | 22.0 | 728 | 32.0 |
|  | Did not self-report HIV positive | 1008 |  | 723 |  | 1731 |  |
| Experienced stigma in health service settings (n=2275) ^d^ | Never | 1394 | 94.1 | 744 | 93.7 | 2138 | 94.0 |
|  | At least once | 87 | 5.9 | 50 | 6.3 | 137 | 6.0 |
| Experienced stigma in the community (n=2275) ^d^ | Never | 1126 | 76.0 | 679 | 85.5 | 1805 | 79.3 |
|  | At least once | 355 | 24.0 | 115 | 14.5 | 470 | 20.7 |
| Current internalized stigma (n=2275) ^d^ | Don't agree | 1175 | 79.3 | 688 | 86.6 | 1863 | 81.9 |
|  | Agree | 306 | 20.7 | 106 | 13.4 | 412 | 18.1 |
| Perceived stigma in health service settings (n=756) ^e^ | No | 371 | 75.9 | 201 | 75.3 | 572 | 75.7 |
|  | Yes | 118 | 24.1 | 66 | 24.7 | 184 | 24.3 |
| Perceived stigma in the community (n=756) ^e^ | No | 173 | 35.4 | 154 | 57.7 | 327 | 43.3 |
|  | Yes | 316 | 64.6 | 113 | 42.3 | 429 | 56.7 |
| Disclosed HIV status | No | 114 | 7.7 | 88 | 11.1 | 202 | 8.9 |
|  | Yes | 1367 | 92.3 | 706 | 88.9 | 2073 | 91.1 |
|  | Did not self-report HIV positive | 1008 |  | 723 |  | 1731 |  |
| Have you ever hidden your ART pills? ^f^ | No | 849 | 69.8 | 544 | 84.5 | 1393 | 74.9 |
|  | Yes | 368 | 30.2 | 100 | 15.5 | 468 | 25.1 |
|  | Did not self-report HIV positive or  ever start ART | 1272 |  | 873 |  | 2145 |  |

^a^ 4006 of 4815 (83.2%) with complete data on viral suppression at PC24 and marital status, self-reported HIV status, experienced or perceived stigma, disclosure of HIV status and hiding pills at PC0.

^b^ Poor or non-adherence was defined as ‘respondents self-reporting that they had ever started ART but were not currently taking ART, or currently taking ART but had either stopped in the past 12 months, or missed pills in the past seven days’.

^c^ Time of first positive HIV test result was asked only to those who self-reported HIV-positive.

^d^ Stigma items captured by PC-HIV+^SR^.

^e^ Stigma items captured by PC-HIV+^sub^ who completed the extended questionnaire (random 20% sample of the Population Cohort.

^f^ Asked to everyone but those ever started on ART.

| **Table S11.** The association between sociodemographic characteristics, ART adherence and self-reported HIV status at PC12 with viral suppression at PC24 in 21 communities in Zambia and South Africa (n=4006) | | | |
| --- | --- | --- | --- |
| **Variable** | **Categories** | ***% virally suppressed***  ***n*/*N* (%)** | **aRR ^a^ (95% CI)** |
| Country ^b^ | Zambia | 1812/2489 (72.8%) | 1.00 |
|  | South Africa | 1025/1517 (67.6%) | **1.23 (1.15-1.32)** |
| Age group ^c^ | 18-24 | 363/723 (50.2%) | 1.00 |
|  | 25-34 | 1263/1765 (71.6%) | **1.43 (1.32-1.55)** |
|  | 35-44 | 1211/1518 (79.8%) | **1.60 (1.48-1.73)** |
| Sex | Male | 314/528 (59.5%) | 1.00 |
|  | Female | 2523/3478 (72.5%) | **0.92 (0.88-0.95)** |
| ART adherence (n=1853) ^d^ | Yes | 1412/1583 (89.2%) | 1.00 |
|  | No (poor adherence) | 216/270 (80.0%) | **0.90 (0.85-0.95)** |
| Self-Reported HIV status ^e^ | Last test HIV-positive on ART | 1631/1846 (88.4%) | 1.00 |
|  | Last test HIV-positive, but not currently on ART or with no data on ART | 228/429 (53.1%) | **0.62 (0.57-0.68)** |
|  | Last test HIV-negative | 390/788 (49.5%) | **0.61 (0.57-0.66)** |
|  | Don't know HIV status | 197/373 (52.8%) | **0.64 (0.58-0.71)** |
|  | Never tested | 391/570 (68.6%) | **0.79 (0.75-0.84)** |

aRR, adjusted risk ratio; CI, confidence interval; n, number of individuals that are virally suppressed; N, total number of individuals within groups; SR: Self-reported. Bolded, p<0.05.

^a^ Adjusted for age group, sex and community with robust standard errors.

^b^ The aRR is adjusted for age group and sex with robust standard errors.

^c^ Overall p value for age group: p<0.001.

^d^ Poor or non-adherence was defined as ‘respondents self-reporting that they had ever started ART but were not currently taking ART, or currently taking ART but had either stopped in the past 12 months, or missed pills in the past seven days’.

^e^ Overall p value for self-reported HIV testing and treatment status: p<0.001.

| **Table S12.** The association between experienced and perceived HIV stigma at PC12 and viral suppression at PC24 in 21 communities in Zambia and South Africa | | | | |
| --- | --- | --- | --- | --- |
| **Variable** | **Categories** | ***n*/*N* (%)** | **aRR ^a^ (95% CI)** | **aRR ^b^ (95% CI)** |
| Self-report HIV status (n=4006) | Self-reported HIV-positive | 1859/2275 (81.7%) | 1.00 | 1.00 |
|  | Did not self-report HIV-positive | 978/1731 (56.5%) | **0.74 (0.70-0.77)** | **0.74 (0.71-0.78)** ^c^ |
| Any stigma (n=2275) | No stigma experienced | 1258/1547 (81.3%) | 1.00 | 1.00 |
|  | Any stigma experienced | 601/728 (82.6%) | 1.00 (0.96-1.05) | 1.01 (0.96-1.05) |
| Experienced stigma in health service settings (n=2275) | Never | 1752/2138 (81.9%) | 1.00 | 1.00 |
|  | At least once | 107/137 (78.1%) | 0.96 (0.88-1.04) | 0.96 (0.88-1.05) |
| Experienced stigma in the community (n=2275) | Never | 1468/1805 (81.3%) | 1.00 | 1.00 |
|  | At least once | 391/470 (83.2%) | 1.01 (0.96-1.06) | 1.01 (0.96-1.06) |
| Current internalized stigma (n=2275) | Disagree | 1522/1863 (81.7%) | 1.00 | 1.00 |
|  | Agree | 337/412 (81.8%) | 1.00 (0.95-1.05) | 1.00 (0.95-1.05) |
| Disclosed HIV status (n=2275) | No | 145/202 (71.8%) | 1.00 | 1.00 |
|  | Yes | 1714/2073 (82.7%) | **1.13 (1.03-1.24)** | **1.12 (1.03-1.23)** |
|  | Did not self-report HIV-positive | 978/1731 (56.5%) | -- | -- |
| Have you ever hidden your ART pills? (n=2275) | No | 1215/1393 (87.2%) | 1.00 | 1.00 |
|  | Yes | 421/468 (90.0%) | 1.01 (0.97-1.05) | 1.01 (0.97-1.05) |
|  | Self-report HIV-positive but never started ART | 223/414 (53.9%) | -- | -- |
| Population group receiving the extended stigma questionnaire (n=4006) | Not receiving the perceived stigma questions | 2286/3250 (70.3%) | 1.00 | 1.00 |
|  | Random 20% sample of the PC | 551/756 (72.9%) | 1.03 (0.98-1.07) | 1.03 (0.98-1.07) |
| Perceived stigma in health service settings (n=756) ^d^ | No | 422/572 (73.8%) | 1.00 | 1.00 |
|  | Yes | 129/184 (70.1%) | 0.95 (0.86-1.06) | 0.98 (0.89-1.08) |
| Perceived stigma in the community (n=756) ^d^ | No | 237/327 (72.5%) | 1.00 | 1.00 |
|  | Yes | 314/429 (73.2%) | 0.97 (0.89-1.06) | 0.96 (0.88-1.05) |

aRR, adjusted risk ratio; CI, confidence interval; n, number of individuals that are virally suppressed; N, total number of individuals within groups. Bolded, p<0.05.

^a^ Model adjusted for age group, sex, and community with robust standard errors.

^b^ Model adjusted for age group, sex, education, marital status, time of diagnosis and community with robust standard errors.

^c^ Model adjusted for age group, sex, education, marital status and community with robust standard errors.

^d^ Stigma items captured by PLHIV who completed the extended questionnaire (random 20% sample of the Population Cohort).
